# Viewing Complex, Dynamic Scenes “Through the Eyes” of Another Person: The Gaze-Replay Paradigm

**DOI:** 10.1371/journal.pone.0134347

**Published:** 2015-08-07

**Authors:** Jennifer Choe Bush, Peter Christopher Pantelis, Xavier Morin Duchesne, Sebastian Alexander Kagemann, Daniel Patrick Kennedy

**Affiliations:** Psychological and Brain Sciences, Indiana University, Bloomington, Indiana, United States of America; Birkbeck, University of London, UNITED KINGDOM

## Abstract

We present a novel “Gaze-Replay” paradigm that allows the experimenter to directly test how particular patterns of visual input—generated from people’s actual gaze patterns—influence the interpretation of the visual scene. Although this paradigm can potentially be applied across domains, here we applied it specifically to social comprehension. Participants viewed complex, dynamic scenes through a small window displaying only the foveal gaze pattern of a gaze “donor.” This was intended to simulate the donor’s visual selection, such that a participant could effectively view scenes “through the eyes” of another person. Throughout the presentation of scenes presented in this manner, participants completed a social comprehension task, assessing their abilities to recognize complex emotions. The primary aim of the study was to assess the viability of this novel approach by examining whether these Gaze-Replay windowed stimuli contain sufficient and meaningful social information for the viewer to complete this social perceptual and cognitive task. The results of the study suggested this to be the case; participants performed better in the Gaze-Replay condition compared to a temporally disrupted control condition, and compared to when they were provided with no visual input. This approach has great future potential for the exploration of experimental questions aiming to unpack the relationship between visual selection, perception, and cognition.

## Introduction

Because the amount of visual information available to us far exceeds that which we are capable of efficiently processing, we attend to areas of the visual environment that are especially likely to yield useful information [1, 2]. This selection process is incredibly important, as we either gain or miss opportunities to extract information based on where we direct our visual attention. To make the most of these opportunities, humans have evolved to use eye movements to fixate the area of highest retinal resolution (i.e., the fovea) on the most important locations in the visual scene.

Visual selection is determined both by reflexive, bottom-up mechanisms (based on low-level features like color, intensity, and orientation) [3] and by slower, top-down, goal-directed processes [4, 5]. Whereas much is known regarding how these mechanisms guide gaze and attention, comparatively less is known about how visual selection (that is, gaze) constrains the ability of the perceptual and cognitive systems to extract information from a given scene, and how to best dissociate these processes. Understanding this relationship is especially important for understanding clinical populations who exhibit both atypical patterns of gaze *and* perceptual/cognitive dysfunction. For instance, among individuals with autism spectrum disorder, abnormal patterns of gaze *correlate* with the ability to judge particular emotional expressions from faces [6] and *correlate* with overall social abilities [7, 8]. Yet, the causal nature of these types of relationships remains unclear; it is possible that atypical gaze patterns *cause* downstream deficits in social perceptual and cognitive performance, but it is also possible that atypical gaze patterns *reflect* atypical social perceptual and cognitive processes in this clinical population.

Here, we present a novel technique—which we call the Gaze-Replay method—that allows the experimenter to dissociate these two aspects of visual comprehension (i.e., selection vs. processing). This paper aims to introduce and demonstrate the feasibility of the technique, using complex, dynamic, semi-naturalistic social stimuli to ask how gaze may directly constrain social comprehension. The Gaze-Replay method intends to show one experimental participant visual stimuli that simulate certain aspects of the visual experience of another person—specifically, what the latter person had approximately foveated.

To create these stimuli, we first collected eye-tracking data from gaze “donors” while they viewed an episode of the American sitcom, *The Office*. Next, using these eye-tracking data, we created a windowed stimulus approximating the foveal input of that donor. These windowed stimuli were then replayed for an independent sample of participants, essentially allowing them to vicariously “look through the eyes” of the donor and use this restricted visual experience to answer questions about the social content of the scenes.

The Gaze-Replay paradigm allows the experimenter to ask whether the area immediately surrounding the donor’s fixation contains sufficient visual information such that another viewer seeing only this area can make proficient judgments (in this case, judgments of complex social emotions). Based on previous research, it may be assumed that the donors are fixating on visually meaningful areas of the scene [1, 2]. However, it cannot be assumed that a viewer who is shown the Gaze-Replay stimuli would be able to extract the same meaning from these fixations. The goal of this study was to test how well viewers are able to utilize the visual information derived from the gaze donors to complete a social cognitive task. If the method were proven to be a viable one, it would allow researchers to present windowed stimuli generated from different donors who exhibit different patterns of gaze, in order to directly observe how different visual inputs affect how viewers interpret the scene.

Previous studies have similarly manipulated the amount of visual information presented to a participant via gaze-contingent or mouse-contingent windowed viewing paradigms, to study how people utilize foveal information in isolation from the periphery [9–11]. Yet, these studies differed from the current one in that viewers still maintained control over the visual information they wished to foveate. Similarly, Dalrymple et al. (2011) simulated for neurotypical participants the experience of simultagnosia (a disorder of visual attention where individuals can only see one object at a time) by utilizing a gaze-contingent windowed viewing paradigm to restrict their visual field. But in this case as well, participants were still allowed to choose where in the scene to direct their gaze. By contrast, the Gaze-Replay technique restricts the viewer’s gaze to what had previously been experienced by the gaze donor, uniquely allowing us to measure the direct impact of visual selection on subsequent processing. The aim of this paper was to explore whether participants are indeed able to extract meaningful information when their view is limited to a donor’s gaze pattern.

To test the viability of the method, we generated Gaze-Replay stimuli from eye-tracking data collected from each of 5 gaze donors while they freely-viewed a full episode of a television sitcom. An independent group of participants then viewed these stimuli, which displayed the foveal gaze patterns of the various gaze donors. We ask whether the Gaze-Replay videos conveyed sufficient social information for viewers to infer the mental states of characters in scenes, as compared to when they were shown gaze patterns that had been temporally disrupted, or when they were provided with no visual information at all. Further, we assess preliminarily whether and how individual differences among gaze donors may have affected viewers’ interpretation of the scene. Our results demonstrate the feasibility of this approach, and we discuss the broad potential for future applications of this method.

## Methods

### Participants–Gaze Donors

We recorded the eye movements of 5 adult “gaze donors.” All donors were male and of normal intelligence (Table 1) as measured by the Weschler Abbreviated Scale of Intelligence [12]. Gaze was recorded at 300 Hz using a Tobii TX300 eye tracker while donors freely viewed an episode of the television show *The Office* with sound [13]. Donors were seated 60–65 cm from the screen of the display computer. The video (24 frames per second, 41.3° x 21.8°) was presented on a 23-inch screen with a refresh rate of 60 Hz. The stimulus was chosen because it is naturalistic compared to more commonly used static or laboratory generated dynamic stimuli, and contains rich and often subtle social interactions. This part of the study was approved by the California Institute of Technology institutional review board, and written informed consent was obtained from all participants.

**Table 1:** Gaze Donor Characteristics.

	Sex	Age	FSIQ	PIQ	VIQ
Donor 1	Male	30	109	100	117
Donor 2	Male	27	126	128	118
Donor 3	Male	25	117	109	119
Donor 4	Male	23	94	92	97
Donor 5	Male	23	110	109	109

FSIQ–Full Scale IQ, PIQ–Performance IQ, VIQ–Verbal IQ

### Participants—Viewers

A total of 246 adults (124 males, 122 females) participated in various conditions within this study (M_age_ = 20.95, SD = 3.41). Participants included Indiana University students and staff members. One hundred seventy-five received course credit for their participation and 71 were compensated at a rate of $15/hr. The study was approved by the Indiana University Institutional Review Board, and written informed consent was obtained from all participants.

### Stimulus Creation

Donors’ eye tracking data were used to generate new stimuli using MATLAB and Psychophysics Toolbox (Version 3.0.10) [14]. First, sound was removed from the original stimulus. Next, a filter was applied to the original stimulus such that, at each frame, only pixels within 3° of the donor’s fixation point (< 128.6 pixels at a viewing distance of approximately 60 cm) remained visible, with the rest of the stimulus blacked out. Three degrees of visual angle was selected because foveal vision encompasses approximately 5°, with the area of highest acuity at the foveola (the center of the fovea, which only contains cone cells) of approximately 1°. This gave us a range of possible window sizes that could be used to approximately convey foveal visual input. Through piloting, we found that a 3° window resulted in a reasonable level of difficulty in performing our specific experimental task.

Pilot testing also suggested that asking participants to continually follow this circular window as it moved about the screen created additional task demands for viewers. Without eye tracking the viewer, one could not be confident that the viewer would be receiving precisely the same foveal input that the gaze donor had, due to saccadic latencies when making unexpected saccades at unexpected times. To eliminate these issues, we presented the window at the center of the screen throughout the video. Thus, the viewer could fixate on a stationary window that dynamically revealed different parts of the original scene.

**Fig 1:**
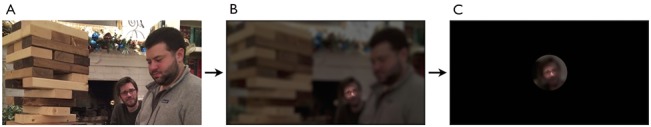
Stimulus Creation. (A) Donor gaze was collected while donors passively viewed dynamic naturalistic stimuli. (B) A filter was then applied to each frame to create a window only exposing 3° of the donor’s fixation point. (C) An example of the final Gaze-Replay windowed stimulus. Examples depicted are for illustration purposes only. The actual stimuli included scenes from the television show, *The Office*.

To add some additional but minimal visual context, we expanded the window with an additional 4° of blurred and dimmed peripheral information. We used alpha blending to make the periphery slightly visible through the black background (16% visible, 84% opaque). The periphery was also blurred with a 45x45 pixel Gaussian (σ = 15 pixel) kernel. Thus the final stimuli consisted of 5 Gaze-Replay windowed (GW) videos designed to approximate the foveal gaze pattern of each respective gaze donor (Fig 1). Each video was then divided into 40 segments (“clips”) each 10–30 seconds in duration.

Although we decided to center the gaze-replay window in the primary GW condition for the aforementioned reasons, we also created a moving Gaze-Replay window (MW) condition, in which the location of the window conveying the gaze of the donor directly matched where the donor was fixating spatially. This condition allowed us to test whether the spatial disruption caused by the centering of the GW condition would affect participant performance.

People are able to differentiate between emotional and neutral stimuli 120–150 ms after stimulus onset [15], and process emotions even when not necessarily consciously aware of the stimulus [16]. Given these remarkable capabilities, we deemed it possible that viewers of the GW stimuli could comprehend the social interactions within a scene from any arbitrary window of visual information, and not only those derived from actual human gaze patterns. Therefore, for comparison, a random window (RW) condition was generated by temporally mismatching the gaze window with respect to the underlying video by 10 minutes. These misaligned stimuli maintained the oculomotor qualities of the true gaze patterns (e.g. the “random” gazes were identical to the true gaze patterns in terms of number, distance, and duration of saccades, fixation characteristics, etc.), but were no longer directed at the same areas of the scene because of this time delay. The random gazes also conveyed the same total amount of visual information. This experimental condition allowed us to examine whether the visual information at the areas of the scene corresponding to the true gaze pattern of the donor contributed to the viewer’s understanding of the scene above and beyond what could have been gleaned from more random visual inputs.

Full-view (FV) and no-view (NV) conditions were also created to respectively assess optimal (ceiling) and chance (baseline) performance on the task. In the FV condition, participants viewed the 40 video clips, with no sound, in their unmodified form. In the NV condition, subjects were asked to perform the emotion recognition task (see below) without seeing any accompanying video clips. This condition was added to establish baseline level performance because it was deemed possible that the familiar nature of the stimuli or general biases toward certain emotions could cause performance to be above statistical chance.

Thus, the final pool of stimuli employed in this study consisted of 200 GW (40 clips x 5 gaze donors), 200 MW, 200 RW, and 40 FV clips.

### Emotion Recognition Task

Participants completed an emotion recognition task, consisting of 40 trials (along with an initial practice trial, which was not analyzed). During the GW, MW, RW and FV conditions, participants viewed a short video clip and were then asked to identify the complex emotion (e.g., disappointed, satisfied, uncomfortable—not limited to the basic emotions of happy, sad, angry, etc.) of a target character in the scene from among 5 choices. The form of each multiple-choice question was identical (i.e., “How is <name of target character> feeling at the end of the clip?”), but the multiple-choice options differed for each trial. No audio was played for any video clip.

**Fig 2:**
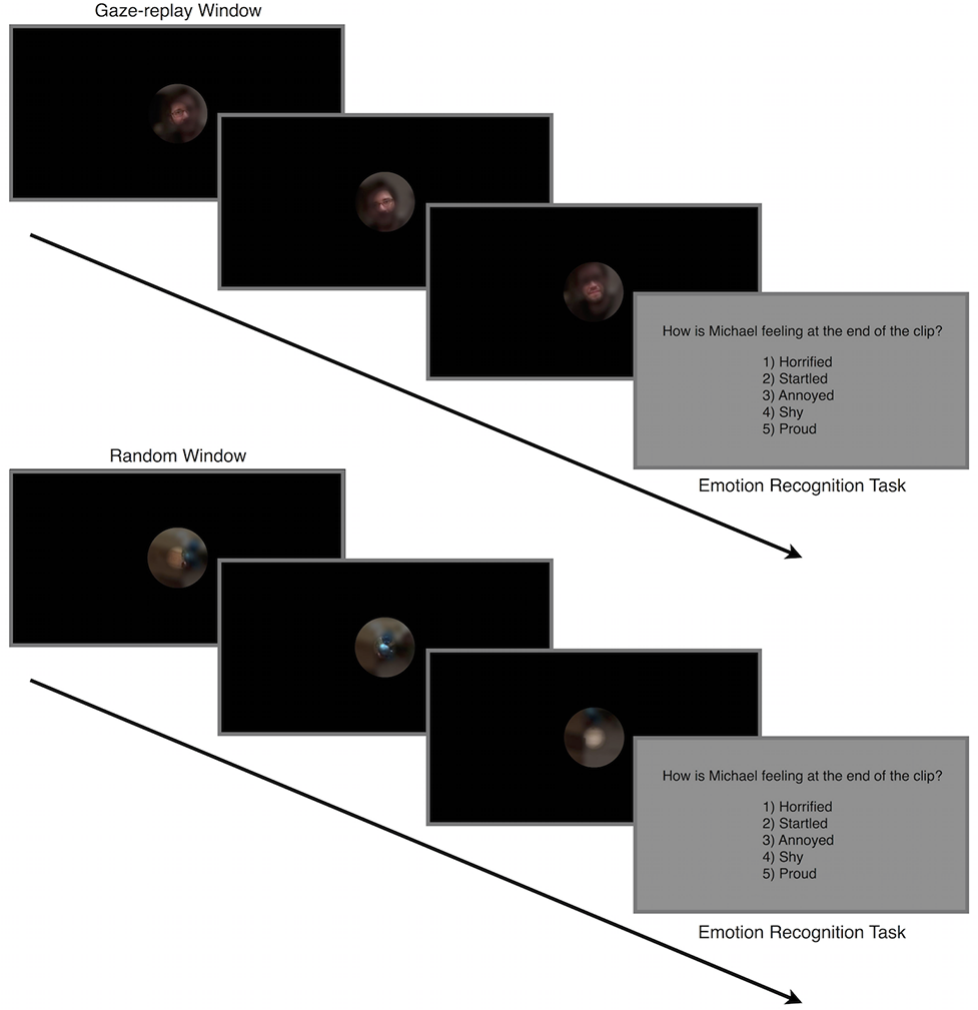
Emotion Recognition Task Procedure. Participants viewed clips in either full-view, gaze-replay window, or random window conditions, or viewed no videos before completing the emotion recognition task. Depicted are examples of gaze-replay window and random window conditions of the same clip. Examples depicted are for illustration purposes only. The actual stimuli included scenes from the television show, *The Office*.

Questions appeared in black text over a gray background. Participants were instructed to press “1”, “2”, “3”, “4”, or “5” on the keyboard to indicate their response, followed by the spacebar to proceed to the next clip (Fig 2). Task items were presented in a randomized order, and there was no enforced time limit for responding to each item (though most participants completed the 40 items in 15–20 minutes).

During the NV condition, participants completed the same emotion recognition task without viewing the video clips. Participants were instructed to select the answer (via mouse click) that would most likely reflect how the target character might feel at any given moment throughout the course of an episode of the show, based on what they might already know about the characters. The form of each multiple-choice question for this condition was modified slightly (i.e. “How does <name of target character> feel?”). However, the multiple-choice options were identical to those offered in the other conditions. The NV condition established baseline performance, which could plausibly be above statistical chance due to either familiarity with the show and its characters, or due to biases toward certain responses shared across conditions.

All participants were provided with a character guide with pictures and names of the characters as well as a glossary containing the definitions of all answer choices and were encouraged to use both at any point during the task. No feedback on performance was provided.

For the FV and NV conditions, all 40 trials were included in the present analyses. For the GW, MW, and RW conditions, half of the trials that participants saw used windows derived from a separate group of gaze donors; these data are not included in the current analyses. Therefore, for GW, MW, and RW conditions, only 20 trials were included for each participant, which were randomly selected among the 40 trials and among the 5 possible gaze donors used to generate the windowed stimuli. The NV group completed the emotion recognition task without watching any videos.

### Procedure—Viewers

Participants were tested in a quiet room for approximately 1 hour in total. The emotion recognition task was presented via MATLAB with Psychophysics Toolbox (Version 3.0.10) [14] for the FV, GW, RW, and MW conditions. The emotion recognition task was presented using Qualtrics software [17] for the NV condition. Participants sat approximately 60–65 cm from a 27-inch screen with a refresh rate of 60 Hz. 58 participants performed only the full-view (FV) condition (34 males, 24 females, M_age_ = 20.3[2.5]). 108 participants performed the gaze-window (GW) condition, followed by the FV condition (53 males, 54 females, M_age_ = 22.0[4.1]). Thirty-four participants performed the random-window (RW) condition, followed by the FV condition (13 males, 21 females, M_age_ = 19.4[0.8]). Thirty participants performed the moving gaze-window (MW) condition, followed by the FV condition (10 males, 20 female, M_age_ = 19.9[1.3]). Seventeen participants answered the questions without video clips (i.e. no-video [NV]; 13 males, 4 females, M_age_ = 19.0[0.8]).

### Procedure–Gaze Donors (Revisited)

Approximately two years after the original gaze data were recorded via eye tracker, all gaze donors were invited back into the laboratory to complete the emotion recognition task in the FV condition. Four out of 5 donors were successfully recruited. Donors were provided with the same instructions, character guide, and word glossaries, and sat approximately 60–65 cm from a 23-inch screen with a refresh rate of 60 Hz. Using these data, we were able to investigate whether there was a relationship between a gaze donor’s competence on the emotion recognition task, and how other participants would perform on the task when viewing scenes “through their eyes.”

### Calculating Task Accuracy

Emotion recognition task accuracy was calculated with respect to the distribution of responses provided by the 58 subjects who only participated in the FV experiment, whom we treated as a normative sample. The accuracy “points” awarded for a given response to a question were calculated by dividing the number of people in the normative sample who had chosen that response by the number of people in the normative sample who had chosen the mode response. For example: A participant is asked, “How is Michael feeling at the end of the clip?” and selects “Embarrassed.” If “Embarrassed” were the mode response for that question because 40 out of the 58 people in the normative sample chose that response, the participant would be awarded 1 point (i.e. 40/40). But if the participant responded “Playful” for that same question, and only 10 people in the normative sample had responded “Playful”, the participant would be awarded 0.25 points (i.e. 10/40).

Thus, a response of “Embarrassed” would be considered to be four times as good as a response of “Playful” for this question, because four times as many participants in the normative, full-view sample had agreed with it. And by setting the denominator to the number of normative participants agreeing with the mode, ceiling performance for the task was normalized to 100%—possible only if the experimental participant selected the mode response for every test item.

### Questionnaires

At the conclusion of the emotion recognition task, all participants completed questionnaires administered using Qualtrics software [17]. Participants completed the Reading the Mind in the Eyes (RME) task [18] as an independent measure of their ability to infer emotion from facial cues. During the administration of RME, participants were provided with a glossary containing the definitions of all answer choices, which they were encouraged to use throughout the task as needed.

In addition, we acquired a background questionnaire assessing their general familiarity with *The Office*, evaluated by how many episodes they had previously seen, whether they had previously seen the specific episode used to derive the stimuli for this study, and how familiar they were with the specific episode on a scale of 1 to 9.

## Results

**Fig 3:**
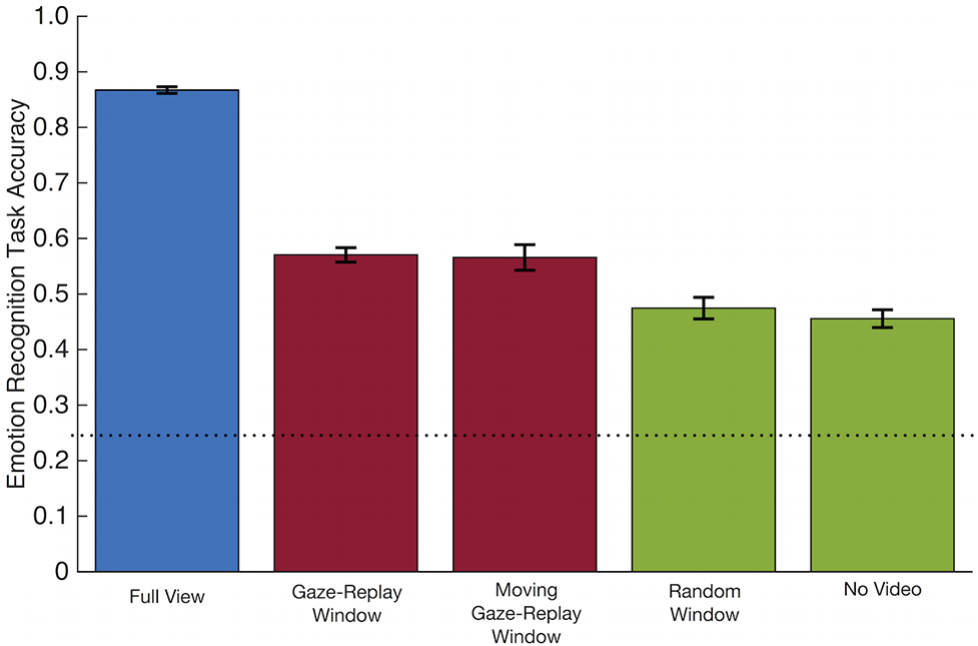
Emotion recognition task accuracy by condition. Conditions represented by different colors are significantly different from each other at *p* < 0.001. Error bars represent standard error. Dotted line depicts chance.

For the GW+FV, RW+FV, MW+FV, and NV groups, participant emotion recognition accuracy was calculated with respect to the normative distribution obtained from the responses provided by the independent sample of FV-only participants (see *Calculating Task Accuracy*). By this measure, the GW+FV, RW+FV, and MW+FV participants performed at 86.7% (SD = 7.7%) in the FV condition, 57.1% (SD = 13.5%) in the GW condition, 56.6% (SD = 12.6%) in the MW condition, 47.5% (SD = 11.3%) in the RW condition, and 45.6% (SD = 6.5%) in the NV condition (Fig 3). Performance was significantly better in the FV condition compared to the three respective windowed conditions (GW: t[106] = 25.9, *p* < 0.001, *d* = 5.03; MW: t[29] = 29.0, *p* < 0.001, *d* = 2.41; RW: t[33] = 20.8, *p* < 0.001, *d* = 7.24, within subjects t-tests), as well as compared to the NV condition (t[156] = 22.6, *p* < 0.001, *d* = 3.62, between subjects t-test). A one-way between subjects ANOVA was conducted to compare emotion recognition accuracy among the GW, MW, RW and NV conditions. There was a main effect of condition [F(3,184) = 8.25, *p* < 0.001, η^2^ = 0.12]. There was no difference in performance between the GW and MW conditions (t[135] = 0.17, ns), suggesting the disruption in spatial information by centering the gaze in the GW condition did not cause participants to perform worse than the MW condition. The mean accuracy for GW was greater than both RW and NV conditions (t[139] = 3.75, *p* < 0.001, *d* = 0.64; t[122] = 3.45, *p* < 0.001, *d* = 0.62, respectively) as was the mean accuracy for MW (t[62] = 3.06, *p* < 0.001, *d* = 0.83; t[45] = 3.36, *p* < 0.001, *d* = 1.05, respectively).

**Fig 4:**
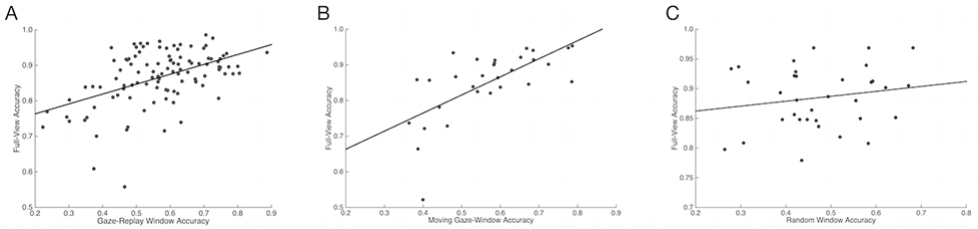
Within subject emotion recognition task accuracy correlations. (A) Strong positive correlation between participants’ full-view and GW accuracies (r[105] = 0.49, *p* < 0.001) (B) Strong positive correlation between participants’ full-view and MW accuracies (r[28] = 0.67, *p* < 0.001) (C) No correlation between full-view and RW accuracies (r[32] = 0.18, ns)

A significant positive correlation was found between performance in GW and FV as well as between MW and FV conditions (r[105] = 0.49, *p* < 0.001; r[28] = 0.67, *p* < 0.001). However, no significant relationship was found for the RW+FV participants between the RW and FV conditions (r[32] = 0.18, ns; Fig 4).

No differences in performance were observed between the RW and NV conditions (t[49] = 0.64, ns). Taken together with the superior performance in GW and MW relative to RW, similar performances in the RW and NV conditions suggest the temporal disruption introduced in the random window condition rendered the visual information useless for the purposes of the emotion recognition task. However, participants performed significantly above chance in both conditions (by our weighted accuracy measure, chance performance would be ~24%).

**Fig 5:**
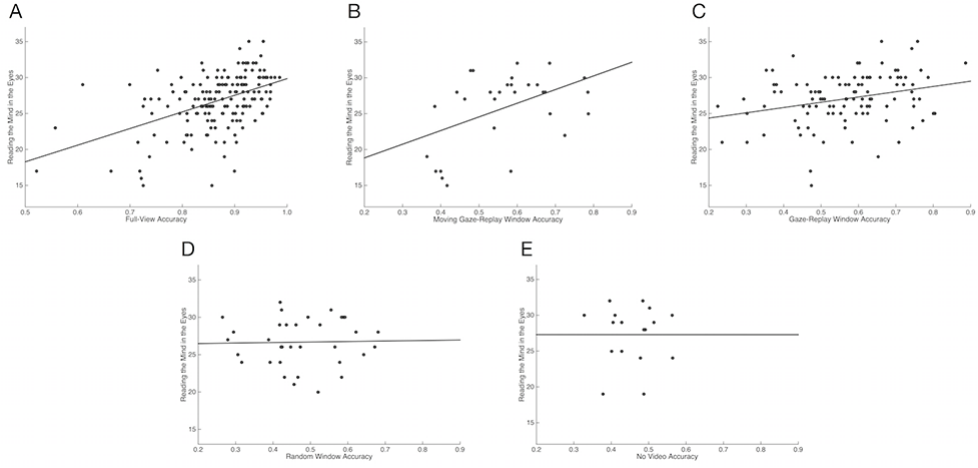
Scatter plot between emotion recognition task accuracy by condition and RME. (A) Strong positive correlation between FV and RME performance (r[139] = 0.45, *p* < 0.001) (B) Weak positive correlation between GW and RME performance (r[105] = 0.28, *p* < 0.001) (C) Strong positive correlation between MW and RME performance (r[28] = 0.47, *p* = 0.01) (D) No correlation between RW and RME performance (r[32] = 0.02, ns) (E) No correlation between NV and RME performance (r[15] = 0, ns)

RME performance overall (M = 26.8, SD = 3.8) was comparable to the mean performance in the general population (26.2) originally reported by Baron-Cohen et al. (2001), and no differences in RME performance were found among the groups assigned to the various conditions (GW+FV, MW + FV, RW+FV, and NV; F[3,187] = 0.99, ns). Emotion recognition accuracies in the FV, GW, and MW conditions were positively correlated with RME performance (r[139] = .45, *p* < 0.001; r[105] = .28, *p* < 0.001; r[28] = .47, *p* = 0.01, respectively). However, performance in the RW and NV conditions were not correlated with RME performance (r[32] = .02, ns; r[15] = .00, ns, respectively; Fig 5).

Participants’ familiarity with the show and with the episode used to create the stimuli was analyzed with respect to task performance. Twenty-seven participants in the GW+FV group reported having seen the episode before, 17 reported they may have, and 63 reported they had not. Eight participants in the MW+FV group had seen the episode, 3 reported they may have, and 19 reported they had not. In the RW+FV group, 12 had seen the episode, 9 reported that they may have, and 19 reported they had not. Overall, the groups did not differ in their familiarity with the particular episode viewed [F(2,168) = 0.11, ns] or with the show in general [F(3,184) = 0.77, ns]. Additionally, using the continuous familiarity ratings acquired, we found familiarity of the episode to be positively correlated with performance in the GW (r[105] = 0.23, *p* = 0.02), MW (r[28] = 0.64, *p* < 0.001), FV (r[169] = 0.30, *p* < 0.001) conditions and trends in the same direction for the RW condition (r[32] = 0.29, *p* = 0.09). Familiarity with the show was not correlated with performance in the NV condition (r[15] = -0.05, ns). Previously discussed results still held when the data were reanalyzed with only participants who reported they had never seen the episode.

**Fig 6:**
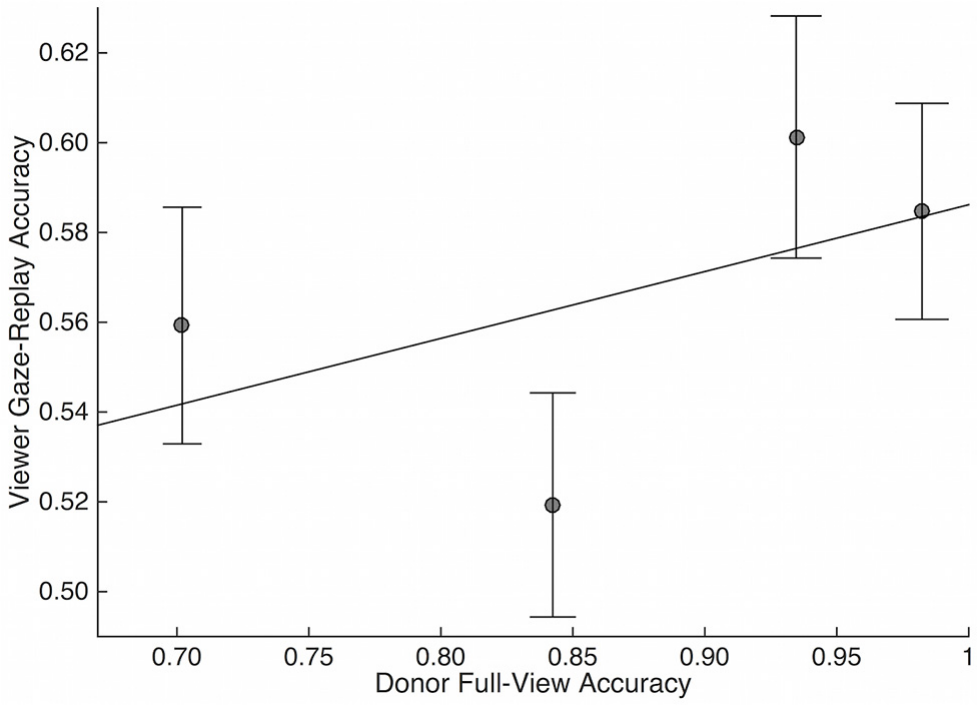
Correlation between donor full-view and viewer gaze-replay performance. Viewer performance in GW condition was correlated with the donor’s full-view performance. However, results were not significant given small sample size. Error bars represent standard error.

Gaze donors’ FV emotion recognition task performance (Donor 1 = 71%, Donor 2 = 93%, Donor 3 = 98%, Donor 4 = 84%) and RME scores (Donor 1 = 25, Donor 2 = 32, Donor 3 = 31, Donor 4 = 34) all fell within the typical ranges of the participants. Lastly, we assessed the relationship between donor’s performance and viewer’s performance on the task when completed with that donor’s GW stimulus. Though there appeared to be a possible positive relationship between donor performance and participants’ associated GW performance (Fig 6), ANOVA did not reveal a significant effect of gaze donor (i.e. which donor was used to generate particular gaze-replay stimuli) on participants’ GW performance (F[4,529] = 1.68, ns).

## Discussion

Our primary aim was to present a novel experimental method that allows naïve viewers to view a visual scene “through the eyes” of another person. To assess the viability of this approach, we tested whether naïve viewers were able to utilize the information conveyed in the “replayed” foveal gaze patterns of multiple donors to make judgments about social exchanges among characters in a television program. The results of this study highlight the potential of this approach for studying the causal relationship between the allocation of visual attention in dynamic scenes and the extraction of information from them (in this case, social information).

Non-foveal, peripheral information was significantly limited in the centered gaze-window (GW), moving gaze-window (MW), and random window (RW) conditions. However, participants were able to identify the complex emotion depicted by the target character significantly better in the GW and MW conditions compared to the RW or NV conditions. This suggests that despite the restricted visual input, meaningful social information was conveyed by donors’ gaze patterns and was successfully utilized by viewers. The RW clips, on the other hand, apparently did not convey any meaningful social information to support task performance; when viewing these clips, subjects’ performance dropped to the same level as having no visual input at all (i.e. NV condition). As expected, participants performed significantly better during the full-view (FV) compared to the other conditions. This particular finding was not surprising given that 1) this was the only condition for which the visual information was unrestricted, and 2) performance in all other conditions was calibrated with respect to the responses of a normative sample of subjects who had viewed the scenes in this same (FV) format. Together, these results support the viability of this method as they provide evidence that viewers are able to utilize visual information derived from gaze donors to successfully complete a social cognitive task.

We found positive correlations between a subject’s emotion recognition accuracy in the FV condition and his or her performance in either the GW or MW condition. We also found correlations between RME scores and FV, GW, and MW accuracies (respectively). In other words, participants’ task accuracy when viewing the replayed gaze was predicted by their social abilities as measured by both RME and performance in FV conditions. However, these correlations were not found in the RW and NV conditions. Thus, the superior social abilities of some participants apparently did not transfer to performance in the RW and NV conditions, probably because these conditions did not contain the social information necessary to bring these abilities to bear on the task.

Although participants were given no visual input in the NV condition, performance in this condition was significantly above chance and similar to performance in the RW condition. We initially hypothesized that participants may have brought prior familiarity with *The Office* to bear on this task; however, no correlation was found between the number of episodes participants reportedly had seen prior to participation and their performance in the experiment., Despite a lack of correlation, one possibility is that subjects used their own intuition about the nature of television shows, or this show specifically, to push their performance above chance. It is also possible that participants were biased toward particular emotional judgments, leading to scores above chance. Thus, these somewhat unexpected results in the NV condition may reflect idiosyncrasies of the stimuli and response choices we employed for this task, but do not detract from our results demonstrating the potential of the Gaze-replay methodology.

Although not statistically significant due to the small sample of gaze donors, we also found preliminary evidence for a positive relationship between a donor’s performance and the performance of participants viewing scenes through that particular donor’s gaze-replay window. In other words, the data suggests it is possible viewers perform better when “looking through the eyes” of a donor who also performed well on the task. This preliminary result not only replicates previous findings that have suggested a relationship between the way in which a person visually explores a scene and how they process it [6, 19] but extends it in a critical way; namely, that the social interpretation of a naïve viewer using the same gaze information can be affected. This approach can be used to identify the causal relationship between gaze and interpretation/social understanding, and may be particularly useful in its application to understanding social deficits in individuals with differences in gaze to social stimuli (e.g., individuals with autism spectrum disorders). While future studies using a larger sample size of donors with well-quantified gaze differences or comprehension are necessary to further understand the relationship between gaze and comprehension on this and other tasks, taken together, the results of this study highlight the importance of gaze for emotion recognition.

Though the current study introduces a valuable new methodology for studying the relationship between gaze and social cognition, it is not without limitations. First, for this experiment, the eye-tracking data of gaze donors were collected while they freely viewed the entire video, but the participants viewed multiple segmented clips while completing a task. We collected gaze data from donors under no task constraints because we wanted to test how the gaze pattern of the donor under naturalistic conditions would influence a naïve viewer’s social comprehension, but different experimental designs can easily be imagined. Second, the eye-tracking data was collected while gaze donors watched the episode with sound, but the experimental task was administered without sound to participants. We made this choice because pilot subjects performed at ceiling even when they were only given audio input from the clips; thus, presenting clips with sound would have made any effect of visual format (GW, MW, RW, or FV) impossible to detect. These methodological choices complicate the interpretation of results, as gaze patterns will be different depending on task demands [20–22] or the presence (or absence) of sound [23, 24]. For example, with the introduction of audio information within a dynamic scene (i.e. a conversation between characters), observers follow the speech turn-taking more closely [24], and look less at the background [23]. Thus, greater effort to maintain similar conditions for both gaze acquisition and gaze-replay testing could be desirable in the design of future experiments.

Eliminating the majority of peripheral information and placing the gaze window in the middle of the screen caused the viewers’ visual experience to be dissimilar from the gaze donor’s actual visual experience in notable ways. Some participants reported the windowed stimuli to be a bit disorienting because of the lack of contextual cues as a result of limited periphery. Although it should be noted that this paradigm is not intended to simulate the true visual experience of the donor with perfect fidelity, we postulate that because people make eye movements to areas that are behaviorally relevant, the gaze-replay window does in fact replicate meaningful qualities of the visual selection and perceptual experience of the donor. The purpose of this study was to ask whether other viewers could exploit the information located in this area of the visual field, even in the absence of additional cues. Despite limitations noted above, our results suggest that this paradigm can successfully address this question. People performed better with donors’ gaze patterns compared to temporally disrupted gaze patterns, confirming that the selection of visual information is important for the extraction of social information.

We selected our stimuli from *The Office* because this television program provides a wide range of facial expressions, face sizes, and number of characters visible across scenes, emulating our real life experiences. However, because we did not systematically generate the scenes with respect to variables like these, the proportional amount of social information varied by frame. The size of the window remained constant throughout the task; thus, when faces were small (i.e. camera zoomed out) they may have fit entirely within the window, whereas only parts of faces were visible when faces were larger (e.g., one eye, or the tip of the nose). It is possible that both the area and the proportion of the faces influenced task performance, with it being easier to identify emotions from smaller faces contained within a window. Future studies may use more uniformly sized faces to utilize this technique to overcome this concern, but this would weaken the ecological validity of the task.

The Gaze-Replay window paradigm could be utilized to study both typical and atypical social perception. In typical development, it could help reveal how gaze patterns affect social understanding and relate to individual differences in social competency. In clinical populations, one could study the directionality of the relationship between gaze and social cognition as well as other possible cascading effects of atypical gaze. For example, one could ask if individuals with autism spectrum disorder (ASD) are not looking at the relevant areas, causing social cognitive deficits, or alternately, if their social comprehension deficits reflect inefficient processing of substantially similar visual input. One could also ask whether social cognition would then improve when ASD individuals are shown the gaze of a neurotypical individual.

The content of scenes used to generate gaze-window stimuli need not be limited to the kind that we selected for this study (i.e., a television program), allowing for the application of this methodology to wide range of stimulus types. Windowed viewing of static faces, for instance, could uncover efficient patterns of visual exploration by task demands. Furthermore, concurrent neuroimaging can be used to investigate the underlying neurological mechanisms that contribute to individual and group differences in social perception. Going beyond the social domain, future applications of the method could also include investigations of how gaze influences other areas of cognition such as memory, attention, preference, and decision-making behaviors.

## Conclusions

We have introduced a novel Gaze-Replay method to examine the relationship between visual selection and subsequent processing of that information. This technique has the potential to be applied to different types of visual stimuli and diverse study populations. The results of this study suggest it can be a valuable tool in the study of social perception and cognition. Participants were able better identify social emotions of characters when shown the foveal gaze pattern of another person through a gaze-replay window compared to when shown a temporally disrupted (essentially random) gaze window or no video. The participant’s own social cognitive abilities predicted their performance on the gaze-replay condition but not the random window or no video, highlighting the influence of both gaze input and an individual’s processing of that gaze on social cognition. We believe the future applications of this method will expand our understanding of the direct consequences of visual selection, with respect to various perceptual and cognitive processes.
